# A multi-model study of the association between BMI and AMH in Chinese women

**DOI:** 10.1530/EC-26-0223

**Published:** 2026-08-05

**Authors:** Zhen Wang, Yanfei Liu, Yonghai Shen, Lin Ma, Xinyan Shi

**Affiliations:** ^1^ Department of Clinical Laboratory, Hangzhou Women’s Hospital, Hangzhou, China; ^2^ Department of Gynecology and Obstetrics, Hangzhou Women’s Hospital, Hangzhou, China

**Keywords:** anti-Müllerian hormone, body mass index, underweight, ovarian reserve, polycystic ovary syndrome, statistical power

## Abstract

**Objective:**

To determine whether body mass index (BMI) is associated with anti-Müllerian hormone (AMH) in Chinese women and whether PCOS, age, or testosterone modifies this association.

**Methods:**

We analyzed 670 women (20–39 years) from Hangzhou Women’s Hospital. Primary analyses included linear regression and generalized additive models (GAMs) with thin-plate splines, and multi-group structural equation modeling to test PCOS effect modification. Secondary analyses included interaction tests and Chinese BMI category comparisons. All models were adjusted for age, follicle-stimulating hormone, luteinizing hormone, and testosterone.

**Results:**

BMI showed no significant linear association with log-AMH (*β* = 0.008; adjusted *R*^2^ = 0.398). GAMs confirmed a near-linear relationship without threshold effects. However, underweight women (BMI < 18.5 kg/m^2^) had significantly lower log-AMH than normal-weight women (*β* = −0.14, *P* = 0.036), with no significant differences for overweight or obese categories. PCOS did not modify the BMI–AMH association.

**Conclusion:**

BMI is not a clinically meaningful predictor of ovarian reserve as a continuous variable. Lower AMH in underweight women highlights a subgroup warranting attention in reproductive assessments, while PCOS status does not influence this relationship.

## Introduction

Anti-Müllerian hormone (AMH) is a dimeric glycoprotein secreted by granulosa cells of growing follicles (1, 2), widely used as a quantitative marker of ovarian reserve (3, 4). Women with polycystic ovary syndrome (PCOS) exhibit two- to three-fold higher circulating AMH than controls (5, 6, 7, 8), yet marked inter-assay heterogeneity limits diagnostic accuracy (8).

The disease prevention bureau of the National Health Commission of China classified overweight as 24.0–28.0 kg/m^2^ and obesity as ≥ 28.0 kg/m^2^ (9). Excess adiposity disrupts the hypothalamic–pituitary–ovarian axis, predisposing to anovulation, menstrual irregularity, and sub-fertility (10, 11, 12). Whether adiposity also erodes ovarian reserve, however, remains contentious. Studies on the body mass index (BMI)–AMH association report conflicting results: some find strong inverse relationships (13, 14), and others find no association (15, 16, 17, 18). This heterogeneity likely reflects three methodological limitations: i) small sample sizes (*n* < 200 in many studies), insufficient to detect modest effects; ii) inadequate confounder adjustment, particularly for age, gonadotrophins, and androgens (16, 19); and iii) arbitrary analytical choices, such as imposing BMI cut-points without validating functional form (20).

To overcome the methodological limitations of prior studies – particularly small sample sizes (*n* < 200 in most cases) and inadequate confounder adjustment – we conducted a large, well-powered study of 670 Chinese women aged 20–39 years. Post hoc power analysis confirmed 80% power to detect a small effect size (Cohen’s *f*^2^ = 0.02) for the BMI–log-AMH association. Within this adequately powered sample, we applied a structured analytical pathway: primary analyses tested core hypotheses using linear regression, generalized additive models (GAMs), and multi-group structural equation modeling; secondary analyses explored categorical effects and interactions; and sensitivity analyses assessed model robustness through influence diagnostics and model comparison. This hierarchical framework ensured that conclusions were driven by pre-specified primary tests rather than post hoc exploration.

## Methods

### Sample size and power considerations

This study included all eligible women attending the hospital during a one-year period (*n* = 670). Post hoc power analysis (G Power 3.1.9.7) indicated 80% power to detect a small effect size (Cohen’s *f*^2^ = 0.02) for the BMI–log-AMH association at *α* = 0.05, with 5 covariates and expected *R*^2^ = 0.35. For the BMI × PCOS interaction, power was 80% to detect *β* = 0.015 (30% slope difference between groups) at *α* = 0.05.

### Study design and participants

This study was a single-center cross-sectional analysis at Hangzhou Women’s Hospital (January–December 2025). The inclusion criteria were as follows: age 20–39 years; reproductive endocrinology clinic attendance; complete data for BMI, AMH, and covariates; and PCOS diagnosed by the 2003 Rotterdam criteria (21). The exclusion criteria were as follows: use of exogenous sex steroids within 3 months, diabetes, hyperandrogenic disorders, systemic infection, inflammatory disease, and incomplete data. Of 712 screened, 670 were included.

### Measurements

Weight and height were measured by calibrated equipment (Seca 703). BMI = kg/m^2^. Serum AMH (electrochemiluminescence, Roche Cobas, Switzerland, e411), follicle-stimulating hormone (FSH), luteinizing hormone (LH), and testosterone (chemiluminescence, Beckman Coulter, USA, DXI800) measured in an ISO15189-certified laboratory.

### Statistical analysis

All analyses were performed using SPSS 26.0 and R 4.4.2. Continuous variables are presented as median (interquartile range), and categorical variables are presented as number (percentage). Two-sided *P* < 0.05 was considered significant. Log-AMH was used as the outcome due to right skew.

### Primary analyses

Primary analyses tested two pre-specified core hypotheses:

Hypothesis 1: Continuous BMI–AMH association. We fitted a linear regression of log-AMH on continuous BMI, adjusted for age, FSH, LH, and testosterone. To validate the linearity assumption without pre-imposing functional form, we additionally fitted a GAM with thin-plate splines (mgcv 1.8–42, *k* = 6, REML). The effective degrees of freedom (EDF) from the GAM served to characterize the shape of the BMI–AMH relationship; EDF ≈ 1 indicates linearity, while EDF > 2 suggests non-linearity.

Hypothesis 2: PCOS effect modification. We used multi-group structural equation modeling (SEM; lavaan 0.6–16) to estimate the BMI–log-AMH association separately in PCOS (*n* = 342) and non-PCOS (*n* = 328) groups. A likelihood-ratio test compared a constrained model (equal BMI coefficients across groups) against an unconstrained model (free coefficients), with a non-significant test indicating no effect modification by PCOS status.

### Secondary analyses

Secondary analyses were exploratory and aimed to generate hypotheses for future testing:

#### Interaction tests

We tested two-way interaction terms (BMI × age and BMI × testosterone) in the linear regression model to assess whether these covariates modified the BMI–AMH association.

#### Chinese BMI category comparison

We classified participants into Chinese categories (underweight: < 18.5, normal weight: 18.5–23.9, overweight: 24.0–27.9, and obese: ≥ 28.0 kg/m^2^) and estimated adjusted marginal means of log-AMH from linear models, with normal weight as the reference. This categorical analysis served to identify potential subgroup deviations that the continuous slope might obscure.

### Sensitivity and robustness analyses

Sensitivity analyses assessed whether primary findings were dependent on model specification or influential observations:

#### Model comparison

We compared the linear, piecewise (23.9 kg/m^2^ threshold), and GAM spline specifications using adjusted *R*^2^, AIC, and BIC. A substantial improvement (ΔAIC > 2 or ΔBIC > 10) in more complex models would challenge the linearity conclusion.

#### Influence diagnostics

We calculated Cook’s distance for each observation and flagged those exceeding the conventional threshold of 4/*n* (0.006). We repeated primary models after excluding influential observations to verify estimate stability.

## Results

### Participant characteristics

Of 670 women, 342 (51%) had PCOS (Table 1).

**Table 1:** Basic information of participants. Data are presented as median (Q1, Q3) or as *n* (%).

	All	BMI				*P*-value
		<18.5	18.5–23.9	24.0–27.9	≥28.0	
*n*	670	113	384	106	67	
Age	29 (26.32)	28 (25.31)	29 (26.33)	30 (26.32)	27 (24.32)	0.109
FSH	6.38 (5.50, 7.56)	7.13 (5.99, 7.89)	6.36 (5.52, 7.42)	5.96 (5.20, 7.36)	6.00 (5.11, 7.07)	0.240
LH	6.31 (4.17, 11.47)	6.0 (4.54, 12.05)	5.79 (3.97, 10.42)	8.77 (4.17, 12.28)	9.65 (5.23, 12.62)	0.034
≤5.9	310 (46.3)	53 (46.9)	198 (51.6)	40 (37.7)	19 (28.4)	
>5.9	360 (53.7)	60 (53.1)	186 (48.4)	66 (62.3)	48 (71.6)	
PRL	11.84 (8.50, 15.50)	11.87 (8.89, 16.25)	11.95 (8.61, 15.84)	11.13 (7.76, 14.10)	11.52 (8.40, 14.05)	0.207
≤15.3	499 (74.5)	81 (71.7)	275 (71.6)	87 (82.1)	54 (80.6)	
>15.3	171 (25.5)	32 (28.3)	109 (28.4)	19 (17.9)	13 (19.4)	
Testosterone	0.56 (0.41, 0.76)	0.49 (0.39, 0.66)	0.53 (0.40, 0.72)	0.64 (0.49, 0.80)	0.76 (0.60, 0.90)	0.000
≤0.75	499 (74.5)	97 (85.8)	302 (78.6)	68 (64.2)	32 (47.8)	
>0.75	171 (25.5)	16 (14.2)	82 (21.4)	38 (35.8)	35 (52.2)	
LH/FSH	1.01 (0.65, 1.84)	0.93 (0.60, 1.73)	0.91 (0.64, 1.65)	1.30 (0.68, 2.12)	1.57 (0.97, 2.09)	0.003
≤0.749	222 (33.1)	41 (36.3)	140 (36.5)	31 (29.2)	10 (14.9)	
>0.749	448 (66.9)	72 (63.7)	244 (63.5)	75 (70.8)	57 (85.1)	
Physical condition						0.000
PCOS	342 (51.04)	34	179	74	55	
Non-PCOS	328 (48.96)	79	205	32	12	
AMH	4.92 (3.07, 8.05)	4.18 (2.72, 6.30)	4.77 (2.86, 7.50)	6.17 (3.68, 9.36)	6.01 (4.02, 9.63)	0.001
<3	162 (24.2)	35 (31.0)	102 (26.6)	17 (16.0)	8 (11.9)	
3-<5	181 (27.0)	37 (32.7)	100 (26.0)	28 (26.4)	16 (23.9)	
5-<9	182 (27.2)	26 (23.0)	104 (27.1)	31 (29.2)	21 (31.3)	
≥9	145 (21.6)	15 (13.3)	78 (20.3)	30 (28.3)	22 (32.8)	

### Primary analyses

#### Continuous BMI–AMH association

BMI showed a weak, non-significant linear association with log-AMH (*β* = 0.008, 95% CI: −0.00–0.02; adjusted *R*^2^ = 0.398) (Table 2). The evidence supports a weak or null linear association.

**Table 2:** Comparison of multiple models and their effect sizes.

Model	Coefficient/metrics	*R* ^2^	AIC/BIC
Linear BMI	0.008 (−0.00 to 0.02)	0.398 (adj)	1,077.14/1,108.69
Piecewise BMI	0.022 (0.00 to 0.04); −0.012 (−0.04 to 0.01)	0.401 (adj)	1,075.33/1,111.39
GAM spline	EDF = 1.16, *P* = 0.179	0.405	1,077.20/1,115.14

#### PCOS effect modification

**Figure 1:**
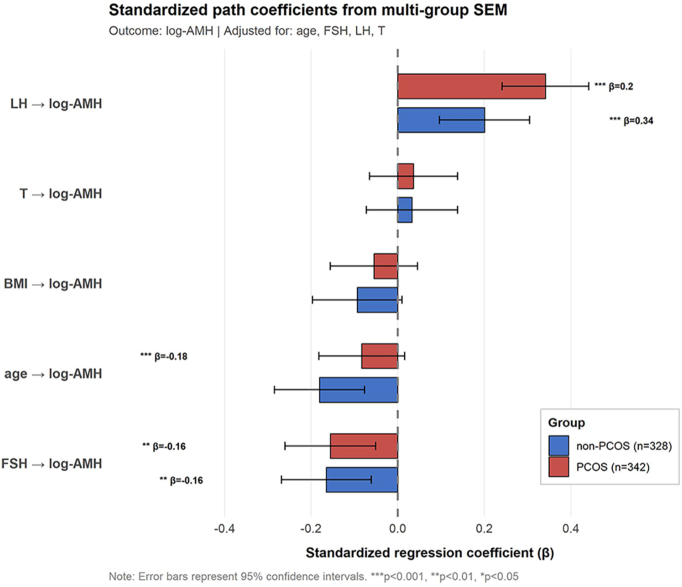
Multi-group structural equation model (SEM): standardized path coefficient (95% confidence interval) of the association between PCOS and non-PCOS female predictors and log-AMH. The model was adjusted for age, FSH, LH, and testosterone (T). ***P < 0.001. Sample size: PCOS group *n* = 342; non-PCOS group *n* = 328. The dashed lines indicate zero effect lines. BMI = body mass index; AMH = anti-Müllerian hormone.

**Figure 2:**
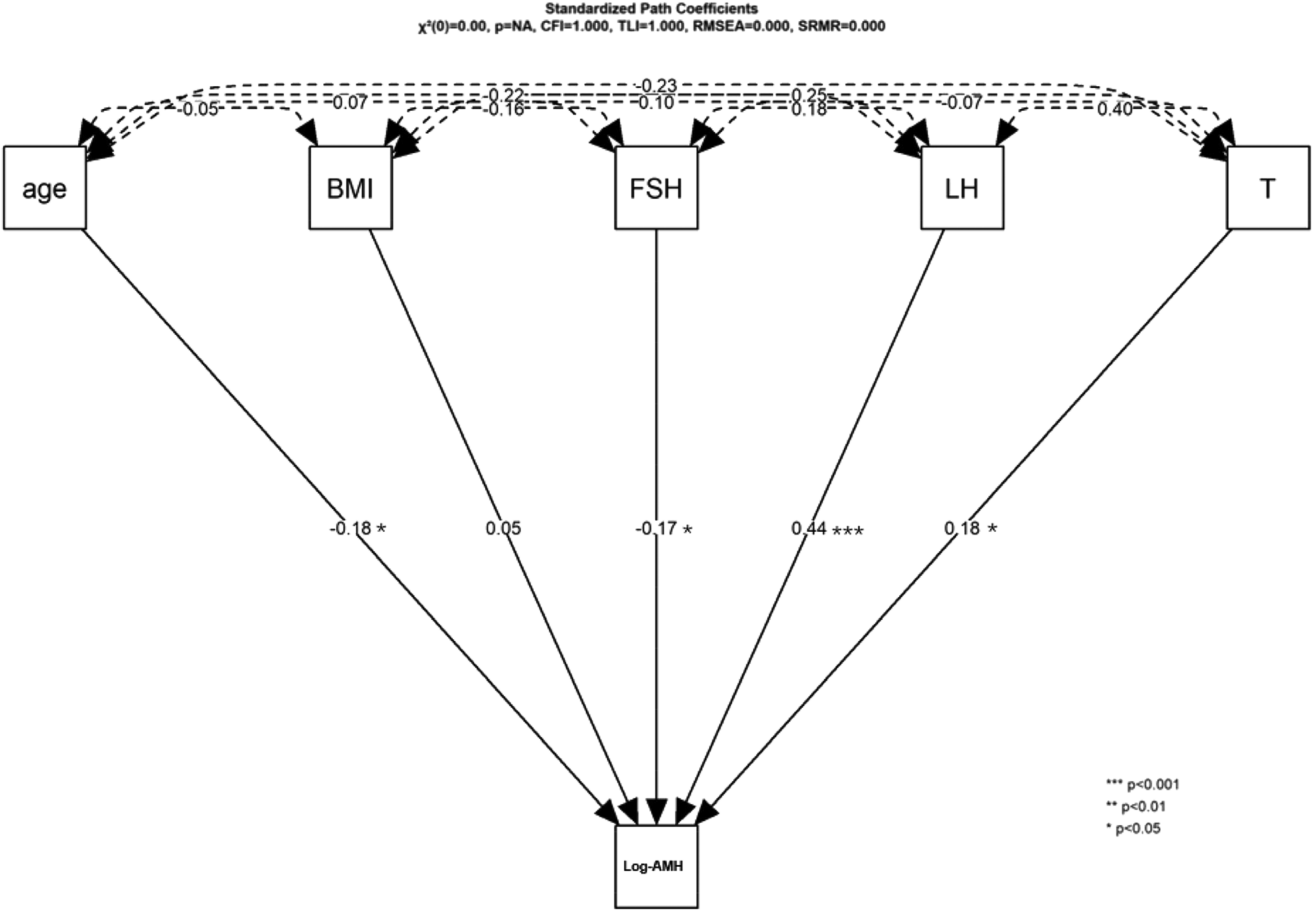
Summary structural equation model path map. Values are standardized estimates. Models were adjusted for age, FSH, LH, and testosterone (T). Fitting index: *χ*^2^(0) = 0.00, *P* = NA, CFI = 1.000, RMSEA = 0.000 (90% CI: 0.02–0.06). BMI = body mass index; AMH = anti-Müllerian hormone.

**Figure 3:**
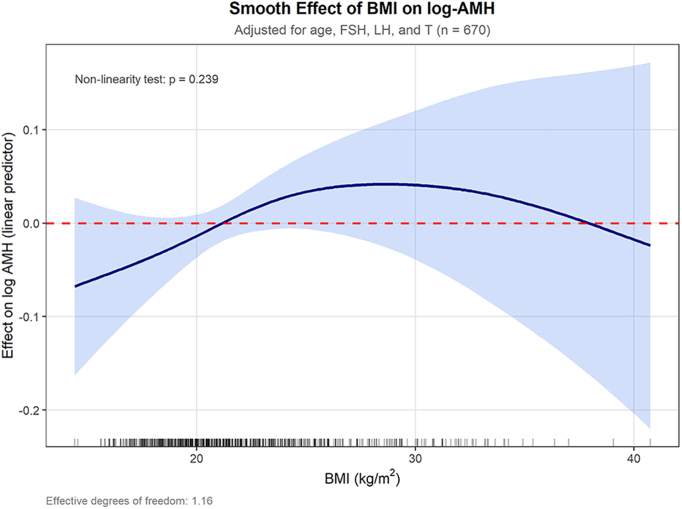
BMI versus log-AMH exposure response curves revealed by GAM. The solid lines represent smoothing function estimates, the light blue shades represent 95% confidence intervals, and the dark blue shades represent 50% confidence intervals. The bottom short vertical bar (rug plot) indicates the BMI data distribution density. Effective degrees of freedom (EDF) = 1.16, indicating an approximate linear relationship. Models were adjusted for age, FSH, LH, and testosterone (T). BMI = body mass index; AMH = anti-Müllerian hormone.

Multi-group SEM showed that BMI was non-significant in both PCOS and non-PCOS groups (95% CIs crossed zero) (Fig. 1). The likelihood-ratio test found no improvement when allowing BMI coefficients to vary by PCOS status, confirming no effect modification. Age (*β* = −0.18, *P* < 0.05), FSH (*β* = −0.17, *P* < 0.05), LH (*β* = 0.44, *P* < 0.001), and testosterone (*β* = 0.18, *P* < 0.05) were significantly associated with log-AMH. Among significant predictors, LH exhibited the strongest standardized effect, followed by age, testosterone, and FSH (Fig. 2). The GAM spline confirmed near-linearity (EDF = 1.16) without threshold effects (Fig. 3).

### Secondary analyses

#### Exploratory interactions

**Figure 4:**
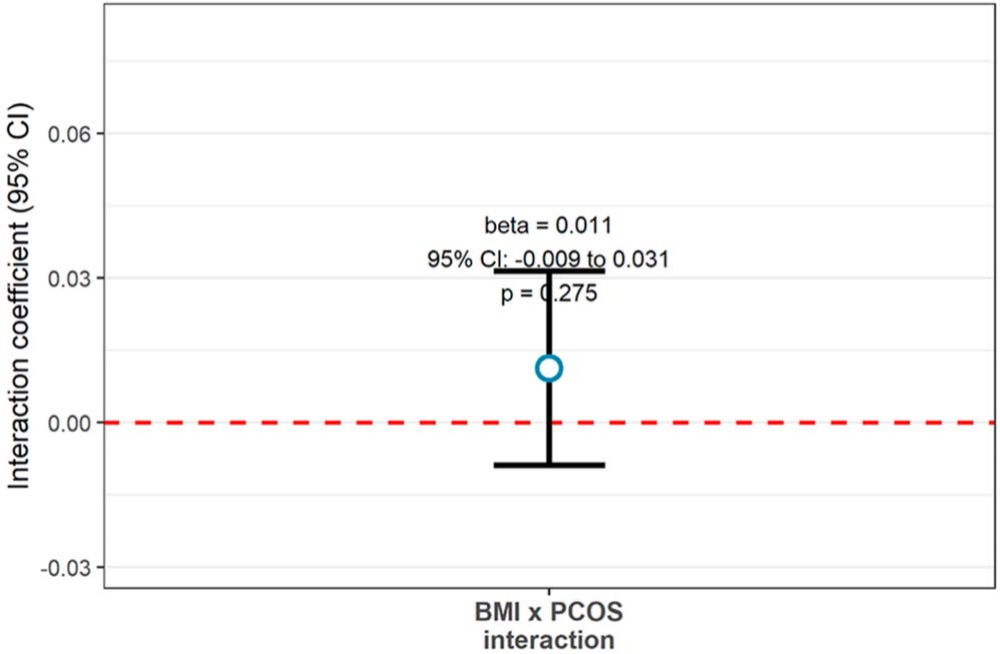
Effect of BMI on log-AMH stratified by PCOS status and interaction test. The forest plot shows the BMI × PCOS interaction coefficient and 95% confidence interval. The interaction was not significant (*β* = 0.011, 95% CI: −0.009–0.031, *P* = 0.275). It suggested that PCOS status did not significantly modify the association between BMI and AMH.

The BMI × PCOS interaction was non-significant (*β* = 0.011, 95% CI: −0.009–0.031, *P* = 0.275) (Fig. 4), corroborating the SEM findings. BMI × age and BMI × testosterone interactions were also non-significant.

#### Chinese BMI category comparison

**Figure 5:**
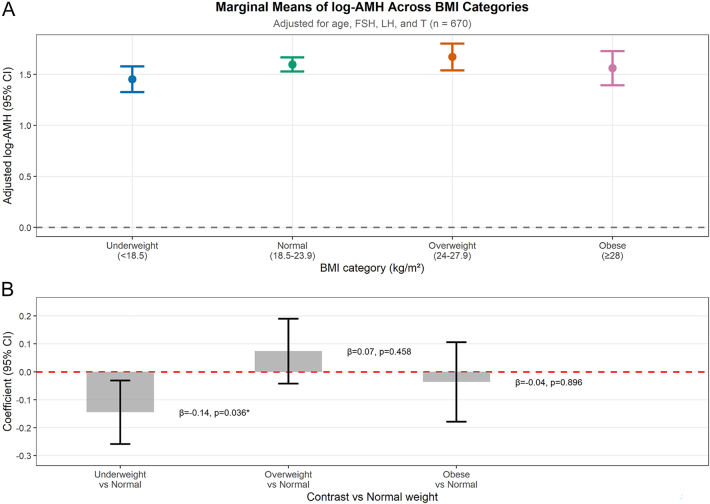
Adjusted marginal mean (95% confidence interval) of log-AMH across BMI categories defined according to the Report on Nutrition and Chronic Disease Status of Chinese Residents (2020) and comparison with the normal-weight group. The model was adjusted for age, FSH, LH, and testosterone (*n* = 670). (A) The dots represent the marginal means after adjusting for age, FSH, LH, and testosterone, and the error bars represent the 95% confidence intervals. Classification criteria: underweight (<18.5 kg/m^2^, *n* = 113), normal weight (18.5–23.9 kg/m^2^, *n* = 384, reference group), overweight (24.0–27.9 kg/m^2^, *n* = 106), and obesity (≥28.0 kg/m^2^, *n* = 67). (B) The forest plot shows the coefficient and 95% confidence interval of each BMI classification compared with the normal-weight group. Only the underweight group showed a significant difference (*β* = −0.14, *P* = 0.036). *P < 0.05. FSH = follicle-stimulating hormone; LH = luteinizing hormone; T = testosterone; BMI = body mass index; AMH = anti-Müllerian hormone.

Compared with normal-weight women, only underweight women had significantly lower log-AMH (*β* = −0.14, *P* = 0.036) (Fig. 5). Overweight (*β* = −0.05, *P* > 0.05) and obese (*β* = −0.01, *P* > 0.05) groups did not differ significantly.

#### Reconciling continuous and categorical findings

The continuous analysis estimates an average slope across the entire BMI distribution, dominated by the normal-weight majority (*n* = 384, 57.3%). The significant underweight effect reflects a deviation in the lower BMI range (*n* = 113, 16.9%) that the linear model does not fully capture, as the slope is pulled toward the null by the large normal-weight group. This does not indicate a non-linear relationship – the GAM EDF = 1.16 confirms linearity – but rather a threshold effect at low BMI that is statistically detectable when this group is isolated from the reference.

### Sensitivity and robustness analyses

#### Model specification

Comparison of linear, piecewise (23.9 kg/m^2^), and GAM spline models showed ΔAIC < 2 and ΔBIC ≤ 15 across specifications (Table 2), indicating that added complexity did not improve fit beyond the linear model.

#### Influence diagnostics

**Figure 6:**
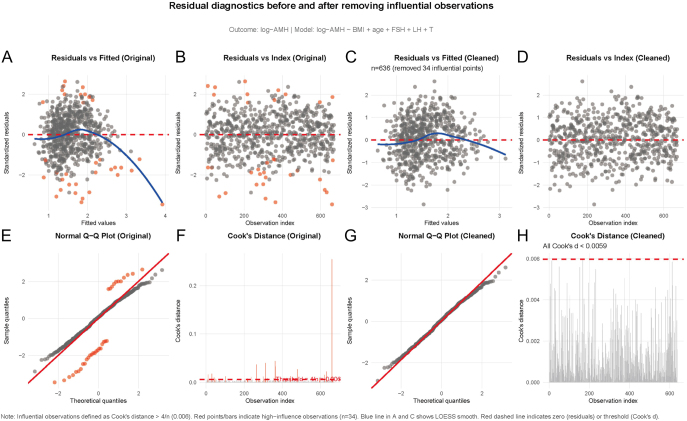
Residual diagnostics of the linear model before and after removing influential observations. (A) Residuals vs Fitted (original): Scatter plot of standardized residuals versus fitted values from the original data. The gray points represent normal observations, the orange points represent influential observations (*n* = 34), the blue curve represents the locally weighted regression (LOESS) smooth, and the red dashed line represents the zero residual line. The plot shows a nonlinear pattern in the original data. (B) Residuals vs Index (original): Scatter plot of standardized residuals versus observation index from the original data. The gray points represent normal observations, the orange points represent influential observations, and the red dashed line represents the zero residual line. (C) Residuals vs Fitted (cleaned): Scatter plot of standardized residuals versus fitted values after removing 34 influential observations (*n* = 636). The gray points represent remaining observations, the blue curve represents the LOESS smooth, and the red dashed line represents the zero residual line. The nonlinear pattern is substantially improved. (D) Residuals vs Index (cleaned): Scatter plot of standardized residuals versus observation index after removing influential observations. Residuals are more evenly distributed. (E) Normal Q–Q plot (original): Normal quantile–quantile plot of standardized residuals from the original data. The gray points represent normal observations, the orange points represent influential observations, and the red line represents the theoretical normal distribution reference line. The plot shows deviation from normality in the tails. (F) Cook’s distance (original): Diagnostic plot of Cook’s distance from the original data. The gray bars represent Cook’s distance for each observation, the red dashed line represents the threshold (Cook’s d = 4/*n* = 0.006), and the orange bars represent influential observations exceeding the threshold. (G) Normal Q–Q plot (cleaned): Normal quantile–quantile plot after removing influential observations. Points are closer to the reference line, indicating improved normality. (H) Cook’s distance (cleaned): Diagnostic plot of Cook’s distance after removing influential observations. All remaining observations have Cook’s distance below the threshold (<0.0059). Note: Influential observations are defined as Cook’s distance > 4/*n* (0.006). The orange points/bars indicate influential observations (*n* = 34). The blue curve in panels A and C shows the LOESS smooth. The red dashed line indicates the zero line (residual plots) or the threshold line (Cook’s distance plots).

Thirty-four observations had Cook’s distance > 0.006. After exclusion, all primary estimates remained stable (Fig. 6), confirming robustness.

## Discussion

### Principal findings

In this large, well-powered study (*n* = 670), we found no significant association between BMI and AMH when analyzed as a continuous variable. The only significant finding was lower AMH in underweight women (BMI < 18.5 kg/m^2^), representing 16.9% of the cohort (*n* = 113). PCOS did not modify the BMI–AMH association. These results were consistent across multiple analytical specifications and robust to outlier exclusion.

### Interpreting the continuous vs categorical discrepancy

A central challenge in interpreting our findings is the apparent tension between the non-significant continuous BMI association and the significant underweight category effect. We propose the following interpretation: the continuous analysis provides the primary estimate. With EDF = 1.16 and ΔAIC < 2 across model specifications, the BMI–AMH relationship is best described as weak and linear across the full distribution. The slope is estimated from the entire cohort and reflects the average change in AMH per unit BMI, which is negligible.

The categorical analysis identifies a specific subgroup deviation. Underweight women (BMI < 18.5 kg/m^2^) represent 16.9% of the cohort. Their lower AMH is a genuine finding that does not alter the overall linear slope because i) the linear model estimates an average effect across the full BMI distribution and the underweight group’s deviation is offset by the predominantly flat relationship in the normal-weight majority; ii) the GAM, which accommodates local curvature without pre-specified cut-points, still yields EDF ≈ 1, confirming no systematic non-linearity; and iii) the effect is most clearly detectable when this group is isolated and compared directly with the reference group.

Thus, the categorical finding does not contradict the continuous analysis – it refines the analysis by identifying a specific at-risk subgroup that the average slope obscures. This is methodologically analogous to detecting an outlier cluster that deviates from an otherwise linear trend.

### Comparison with prior literature

Our findings contrast with studies reporting strong inverse BMI–AMH associations (13, 14) but align with those finding no significant link (15, 16, 17, 18). We attribute this consistency to our large sample size and rigorous adjustments: the previous discovery of a significant negative correlation may have been influenced by smaller sample sizes or differences in confounder adjustment. The structured framework, which distinguishes primary hypothesis testing from secondary exploration, reduces the risk of false positives arising from multiple comparisons.

### Clinical implications

We acknowledge the limited direct clinical relevance of our findings for overweight and obese women, whose management is already guided by established recommendations (10, 11, 12, 22). However, our study offers two nuanced contributions.

First, it clarifies the ovarian reserve perspective. Clinicians counseling patients about weight and fertility often cite BMI–AMH associations as a rationale for weight loss. Our data suggest that for ovarian reserve specifically, BMI is not a strong predictor in Chinese women aged 20–39 years. This does not negate broader metabolic benefits of weight management but cautions against overinterpreting BMI as a determinant of follicle pool size.

Second, it highlights underweight as a potential concern. The significantly lower AMH in underweight women, representing nearly one-sixth of the cohort, is consistent with evidence that extremely low body weight disrupts the hypothalamic–pituitary–ovarian function (10, 11, 12). This may warrant AMH assessment in underweight women presenting with fertility concerns, a group often overlooked in obesity-focused reproductive medicine.

For PCOS specifically, the absence of effect modification suggests that weight management advice need not be stratified by PCOS status for ovarian reserve purposes. The slope of the BMI–AMH relationship is equally flat in both groups.

Strengths include the large sample with confirmed statistical power, structured primary/secondary analysis framework, rigorous confounder adjustment, and multi-method robustness checks.

The limitations of this study are as follows: Cross-sectional design precludes causal inference. Hospital-based recruitment may limit generalizability. Residual confounding by insulin resistance, smoking, diet, or physical activity cannot be excluded. Findings for the underweight group, though based on a sizeable subgroup (*n* = 113), require confirmation in prospective studies. Findings apply to women aged 20–39 years.

## Conclusion

In this well-powered study, BMI was not significantly associated with AMH as a continuous variable. The only significant finding – lower AMH in underweight women – represents a BMI threshold effect at the lower range that does not alter the overall weak, linear relationship across the full distribution. PCOS did not modify this association. These findings suggest that BMI is not a clinically meaningful predictor of ovarian reserve in this population, although underweight status may warrant specific attention. Public health messages should continue to emphasize overall weight management for general health, without implying that BMI thresholds specifically protect ovarian reserve.

## Declaration of interest

The authors declare that the research was conducted in the absence of any commercial or financial relationships that could be construed as a potential conflict of interest.

## Funding

This work was supported by the General Project of Hangzhou Science and Technology Bureauhttps://doi.org/10.13039/501100003786 (202204B16).

## Author contribution statement

ZW performed formal analysis, visualization, data curation, and investigation and wrote the original draft. YL designed the methodology and wrote the original draft. YS and XS wrote, reviewed, and edited the manuscript. LM acquired funding and wrote, reviewed, and edited the manuscript.

## Data availability

The de-identified participant data, analysis code, and output files that support the findings of this study are available upon reasonable request. Data requests should be directed to the corresponding author at 1115yue@163.com.

## Ethical approval and informed consent

The study protocol was formally approved by the Hangzhou Women’s Hospital Institutional Review Board (approval no. 2025-A-154). All participants provided written informed consent after the study purpose, procedures, and potential risks had been fully explained. The conduct of the study adhered to the Declaration of Helsinki and relevant ethical guidelines to ensure participant privacy and data security.

## Consent for publication

All authors have read the final manuscript and confirm that all figures, tables, videos, audio recordings, and any other accompanying materials are accurate and may be published. Written permission has been obtained from any individuals who can be identified in such a material, and all potentially identifying data have been appropriately anonymized.

## References

[1] Dewailly D, Andersen CY, Balen A, et al. The physiology and clinical utility of anti-Müllerian hormone in women. Hum Reprod Update 2014 20 370–385. (10.1093/humupd/dmu043) 24430863

[2] di Clemente N, Racine C, Pierre A, et al. Anti-Müllerian hormone in female reproduction. Endocr Rev 2021 42 753–782. (10.1210/endrev/bnab012) 33851994

[3] Rong L, Huiyu X, Li J, et al. Expert consensus on the clinical application of anti-Müller tube hormones (2023 edition). Chin J Pract Gynecol Obstet 2023 39 431–439. (10.19538/j.fk2023040111)

[4] Harris BS, Jukic AM, Truong T, et al. Markers of ovarian reserve as predictors of future fertility. Fertil Steril 2023 119 99–106. (10.1016/j.fertnstert.2022.10.014) 36460524 PMC10074834

[5] Cook CL, Siow Y, Brenner AG, et al. Relationship between serum Müllerian-inhibiting substance and other reproductive hormones in untreated women with polycystic ovary syndrome and normal women. Fertil Steril 2002 77 141–146. (10.1016/s0015-0282(01)02944-2) 11779604

[6] Eilertsen TB, Vanky E & Carlsen SM. Anti-Mullerian hormone in the diagnosis of polycystic ovary syndrome: can morphologic description be replaced? Hum Reprod 2012 27 2494–2502. (10.1093/humrep/des213) 22693172

[7] Fraissinet A, Robin G, Pigny P, et al. Use of the serum anti-Müllerian hormone assay as a surrogate for polycystic ovarian morphology: impact on diagnosis and phenotypic classification of polycystic ovary syndrome. Hum Reprod 2017 32 1716–1722. (10.1093/humrep/dex239) 28854589

[8] Teede H, Misso M, Tassone EC, et al. Anti-Müllerian hormone in PCOS: a review informing international guidelines. Trends Endocrinol Metab 2019 30 467–478. (10.1016/j.tem.2019.04.006) 31160167

[9] National Health Commission of the People’s Republic of China. Report on Nutrition and Chronic Disease Status of Chinese Residents (2020), pp 1–148. Beijing: People’s Medical Publishing House, 2022.

[10] Rittenberg V, Sobaleva S, Ahmad A, et al. Influence of BMI on risk of miscarriage after single blastocyst transfer. Hum Reprod 2011 26 2642–2650. (10.1093/humrep/der254) 21813669

[11] Russo M, Ates S, Shaulov T, et al. Morbid obesity and pregnancy outcomes after single blastocyst transfer: a retrospective, North American Study. J Assist Reprod Genet 2017 34 451–457. (10.1007/s10815-017-0883-9) 28190215 PMC5401700

[12] van Duijn L, Rousian M, Hoek J, et al. Higher preconceptional maternal body mass index is associated with faster early preimplantation embryonic development: the Rotterdam periconception cohort. Reprod Biol Endocrinol 2021 19 145. (10.1186/s12958-021-00822-0) 34537064 PMC8449446

[13] Kriseman M, Mills C, Kovanci E, et al. Antimullerian hormone levels are inversely associated with body mass index (BMI) in women with polycystic ovary syndrome. J Assist Reprod Genet 2015 32 1313–1316. (10.1007/s10815-015-0540-0) 26238387 PMC4595400

[14] Zhang M, Liu X, Xu X, et al. The reference value of anti-Müllerian hormone to diagnose polycystic ovary syndrome is inversely associated with BMI: a retrospective study. Reprod Biol Endocrinol 2023 21 15. (10.1186/s12958-023-01064-y) 36726106 PMC9890853

[15] Dileep A & Saeed S. Evaluating serum anti‐Müllerian hormone as a diagnostic biomarker for polycystic ovary syndrome in Pakistani women. Int J Gynecol Obstet 2025 171 676–683. (10.1002/ijgo.70240) 40560122

[16] Kloos J, Coyne K & Weinerman R. The relationship between anti‐Müllerian hormone, body mass index and weight loss: a review of the literature. Clin Obes 2022 12 e12559. (10.1111/cob.12559) 36181300 PMC9787654

[17] Pambudi D, Saadi A & Sudjarwo S. Analisis Antimullerian Hormon (AMH) di Dalam Serum pada Berbagai Kategori Indeks Masa Tubuh. J Surya Medika 2019 5 162–168. (10.33084/jsm.v5i1.956)

[18] Woo H-Y, Kim K-H, Rhee E-J, et al. Differences of the association of anti-Müllerian hormone with clinical or biochemical characteristics between women with and without polycystic ovary syndrome. Endocr J 2012 59 781–790. (10.1507/endocrj.ej12-0055) 22673409

[19] Palomaki GE, Kalra B, Kumar T, et al. Adjusting antimüllerian hormone levels for age and body mass index improves detection of polycystic ovary syndrome. Fertil Steril 2020 113 876–884.e2. (10.1016/j.fertnstert.2019.12.012) 32147175 PMC7583345

[20] Guan M, Feng J, Zhu M, et al. Triglyceride glucose index – body mass index predicts insulin resistance, metabolic syndrome and associates with impaired ovulation in Chinese women with polycystic ovary syndrome. Front Endocrinol 2025 16 1653636. (10.3389/fendo.2025.1653636) PMC12488453 41048427

[21] Rotterdam ESHRE/ASRM-Sponsored PCOS Consensus Workshop Group. Revised 2003 consensus on diagnostic criteria and long-term health risks related to polycystic ovary syndrome. Fertil Steril 2004 81 19–25. (10.1016/j.fertnstert.2003.10.004) 14711538

[22] Jaacks LM, Vandevijvere S, Pan A, et al. The obesity transition: stages of the global epidemic. Lancet Diabetes Endocrinol 2019 7 231–240. (10.1016/s2213-8587(19)30026-9) 30704950 PMC7360432

